# Energy metabolism in intestinal epithelial cells during maturation along the crypt-villus axis

**DOI:** 10.1038/srep31917

**Published:** 2016-08-25

**Authors:** Huansheng Yang, Xiaocheng Wang, Xia Xiong, Yulong Yin

**Affiliations:** 1 Animal Nutrition and Human Health Laboratory, School of Life Sciences, Hunan Normal University, Changsha, China; 2 Chinese Academy of Science, Institute of Subtropical Agriculture, Research Center of Healthy Breeding Livestock & Poultry, Human Engineering & Research Center of Animal & Poultry Science, Key Lab Agroecology Processing Subtropical Region, Scientific Observational and Experimental Station of Animal Nutrition and Feed Science in South-Central, Ministry of Agriculture, Changsha, China; 3 Fujian Aonong Bio-Technology Co., Ltd., Xiamen, China

## Abstract

Intestinal epithelial cells continuously migrate and mature along crypt-villus axis (CVA), while the changes in energy metabolism during maturation are unclear in neonates. The present study was conducted to test the hypothesis that the energy metabolism in intestinal epithelial cells would be changed during maturation along CVA in neonates. Eight 21-day-old suckling piglets were used. Intestinal epithelial cells were isolated sequentially along CVA, and proteomics was used to analyze the changes in proteins expression in epithelial cells along CVA. The identified differentially expressed proteins were mainly involved in cellular process, metabolic process, biological regulation, pigmentation, multicellular organizational process and so on. The energy metabolism in intestinal epithelial cells of piglets was increased from the bottom of crypt to the top of villi. Moreover, the expression of proteins related to the metabolism of glucose, most of amino acids, and fatty acids was increased in intestinal epithelial cells during maturation along CVA, while the expression of proteins related to glutamine metabolism was decreased from crypt to villus tip. The expression of proteins involved in citrate cycle was also increased intestinal epithelial cells during maturation along CVA. Moreover, dietary supplementation with different energy sources had different effects on intestinal structure of weaned piglets.

The intestine has a high rate of energy expenditure because the digestion and absorption processes are critically depend on energy^1^. It was reported that the portal-drained viscera (PDV; including intestines, pancreas, spleen, and stomach) contribute less than 6% of body weight, but contribute 20–35% of whole-body energy expenditure^1^,^2^,^3^. Intestinal mucosa energy metabolism is very complex, because the energy source for intestinal mucosa is a complex mixture of arterial and luminal substrates and the pattern of intestinal substrates oxidation can be altered by nutrients composition in the diets^4^. Moreover, besides glucose and fatty acids, amino acids, especially glutamate and glutamine, also play important roles in providing energy to intestinal epithelial cells. Stoll *et al.*^5^ showed that enteral glucose, arterial glutamine, arterial glucose, and enteral glutamate contributed to 15%, 19%, 29% and 36% of the total production of CO2 in the PDV of piglets, respectively^5^. Moreover, about three-fourths of the energy requirement for the PDV were provided by the oxidation of glucose, glutamine, and glutamate^5^.

The mammalian intestine plays a key role in nutrients digestion and absorption and has an impact on the functions of the entire organism. The intestinal lumen is lined by a monolayer of epithelial cells (or mucosa), which perform the primary functions in digesting and absorbing nutrients, and form a barrier against luminal pathogens and toxic substances^6^. The small intestinal epithelium can be divided into two parts, the flask-shaped submucosal invaginations were termed crypts, and the finger-like luminal protrusions were known as villi^7^. These morphologically distinct compartments have different functions in the small intestinal epithelium^7^. The mature epithelial cells covered villi arise from multipotent stem cells, which undergo proliferation and differentiation when they migrate from the crypt compartment^8^,^9^. About 3 days after their terminal differentiation, the mature cells reach villus tip, undergo apoptosis, and are ultimately shed into the intestinal lumen^10^. The continual renewal of epithelial cells along the crypt-villus axis (CVA) ensures the functions of small intestine^7^. It was reported that the metabolism of glucose, fatty acid, and amino acid might be changed in villi compared with crypt^11^,^12^.

Regulation of intestinal mucosal growth and maturation in the neonate is the fundamental issues of mucosal biology because the intestinal mucosa undergoes a very dynamic development process^13^. The mucosal development is dominated by the proliferation and differentiation of epithelial cells^14^. However, studies designed to study the proliferation and differentiation processes of intestinal epithelial cells have been largely conducted using cell culture models, and this area of intestinal mucosal renewal remains poorly understood^15^. Moreover, the limited studies about epithelial cells maturation along CVA were largely performed using adult rodents as animal models, while the epithelial cells lining the intestinal mucosa are different between neonate and adult^7^,^11^,^12^,^16^. The intestinal development and digestive function in rodents are also considerably different from those in man^12^. The swine species is closely comparable with man in intestinal system, and thus considered as an ideal model to investigate intestinal function and development^17^. However, to our knowledge, very few experiments were conducted to study swine small intestinal epithelium maturation along CVA, and these experiments were focused on digestive enzyme activity, nutrients uptake and transporters expression of neonatal pig^18^,^19^,^20^.

In the present experiment, we hypothesized that the energy metabolism in intestinal epithelial cells would be changed during maturation along CVA in neonates. The jejunal epithelial cells were serially isolated along CVA in neonatal pig using a method developed previously^18^. The expression of proteins related energy metabolism in epithelial cells were determined using isobaric mass tags for relative and absolute quantitation (iTRAQ), and the RT-PCR and biochemical analysis were used to confirm it. The results of the present study will reveal a global change in energy metabolism in intestinal epithelial cells along CVA, and provide potential methods to use energy sources to regulate intestinal morphology and functions.

## Resultss

### Changes in proteins expression in intestinal epithelial cells during maturation along CVA

**Figure 1:**
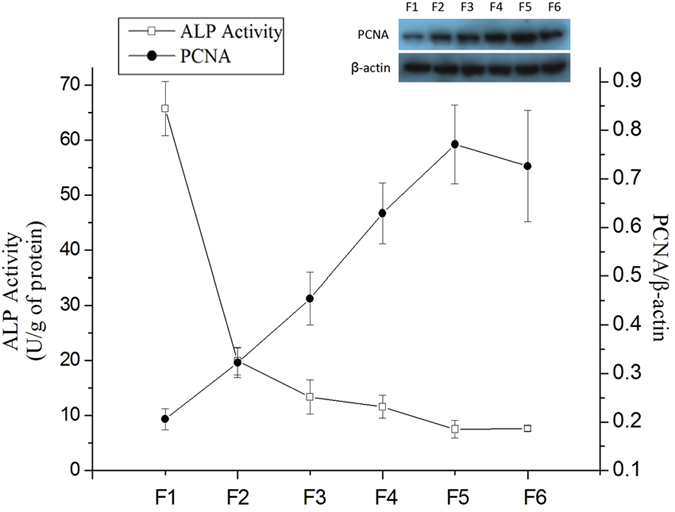
The activity of alkaline phosphatase (ALP) and expression of PCNA along CVA. The activities of ALP in intestinal epithelial cells were measured using commercial kits according to the manufacturer’s protocol. The expression of PCNA was determined using Western blot, and the abundance of PCNA along CVA was normalized using β-actin as an internal control.

**Figure 2:**
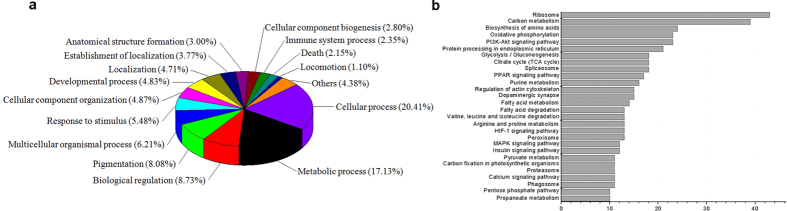
Functional categorization (biological process) and KEGG pathways enrichment of differentially expressed proteins along CVA. Functional categorization (**A**) was performed using WEGO program, and KEGG pathways enrichment (**B**) was performed using on line program (www.kegg.jp).

The intestinal epithelial cells were sequentially isolated from villus tip to crypt bottom and yield 6 “cell fractions” (designated F1 through F6), therefore the maturation process of intestinal epithelial cells goes from F6 (crypt bottom) to F1 (villus tip). The cell fraction procedure was validated by measuring the activity of ALP (marker of intestinal epithelial cells differentiation) and the expression of PCNA (marker of DNA synthesis) along CVA. The activity of ALP decreased from F1 (villus tip) to F6 (crypt bottom), while the expression of PCNA gradually increased from F1 to F5/F6 (Fig. 1). These results indicated that the epithelial cells with different degree of differentiation were successfully isolated from piglet small intestine. A global proteins expression profile in intestinal epithelial cells during maturation along CVA was determined using iTRAQ. A total of 3241 uniqueproteins were identified. Of these, 865 were named and quantified proteins, and 768 proteins differentially expressed along CVA. GO enrichment analysis showed that the differentially expressed proteins were mainly involved in cellular process, metabolic process, biological regulation, pigmentation, multicellular organizational process, response to stimulus, cellular component organization, development process, and localization and so on (Fig. 2A). KEGG pathway enrichment analysis showed the differentially expressed proteins were mainly involved in ribosome, carbon metabolism, oxidative phosphorylation, PI3K-Akt signaling pathway, protein processing in endoplasmic reticulum, glycolysis/gluconeogenesis, citrate cycle (TCA cycle), spliceosome, and PPAR signaling pathway and so on (Fig. 2B).

**Figure 3:**
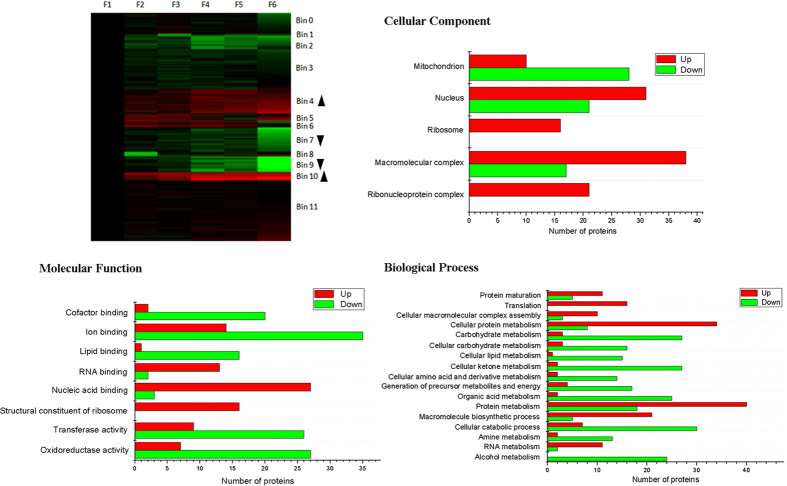
Functional categorization of up-regulated and down-regulated proteins along CVA. The differentially expressed proteins were clustered using Cluster 3.0 program with k-means clustering, then the proteins in up-regulated (Up; Bin 4 and Bin 10) and down-regulated (Down; Bin 7 and Bin 9) clusters were compared using WEGO program.

As seen in Fig. 3, the differentially expressed proteins was found to have clustered into 12 distinct groups (Bin 0 to Bin 11). Among these distinct groups, the expression of proteins in Bin 4 and Bin 10 increased from F1 to F6, while the expression of proteins in Bin 7 and Bin 9 decreased from F1 to F6. The WEGO program was used to analyze GO enrichment of up-regulated proteins (Up; Bin 0 and Bin 10) and down-regulated proteins (Down; Bin 7 and Bin 9), the GO terms that were significantly (p-value < 0.05) different between up-regulated and down-regulated proteins were selected. On the ontology type of cellular component, mitochondrion related proteins in up-regulated were significantly less than in down-regulated proteins, while nucleus, ribosome, macromolecular complex, and ribonucleoprotein complex related proteins in up-regulated proteins were significantly more than in down-regulated proteins. On the ontology type of molecular function, cofactor binding, ion binding, lipid binding, transferase activity, and oxidoreductase activity related proteins in up-regulated proteins were significantly less than in down-regulated proteins; RNA binding, nucleic acid binding, and structural constituent of ribosome related proteins in up-regulated proteins were significantly more than in down-regulated proteins. On the ontology type of molecular function, protein maturation, translation, cellular macromolecular complex assembly, cellular protein metabolism, protein metabolism, macromolecule biosynthetic process, and RNA metabolism related proteins in un-regulated proteins were significantly more than in down-regulated proteins; carbohydrate, cellular carbohydrate, cellular lipid, cellular ketone, cellular amino acid and derivative, organic acid, amine, and alcohol metabolism, generation of precursor metabolites and energy, and cellular catabolic process related proteins in un-regulated proteins were significantly less than in down-regulated proteins.

### Generation of precursor metabolites and energy

**Figure 4:**
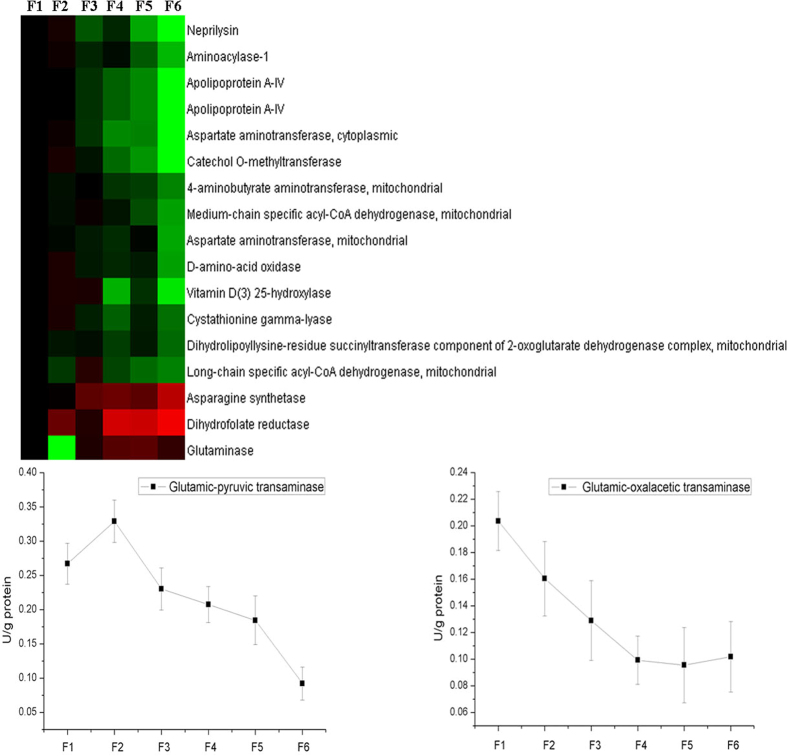
The expression of proteins in generation of precursor metabolites and energy along CVA. The proteins in energy metabolism pathway were selected based on the results of functional categorization.

A total of 21 proteins involved in generation of precursor metabolites and energy were identified from the differentially expressed proteins, with 17 proteins down-regulated in expression from F1 to F6, and these proteins were mainly involved in glycolysis, citrate cycle, and fatty acids metabolism (Fig. 4).

### Glycolysis

**Figure 5:**
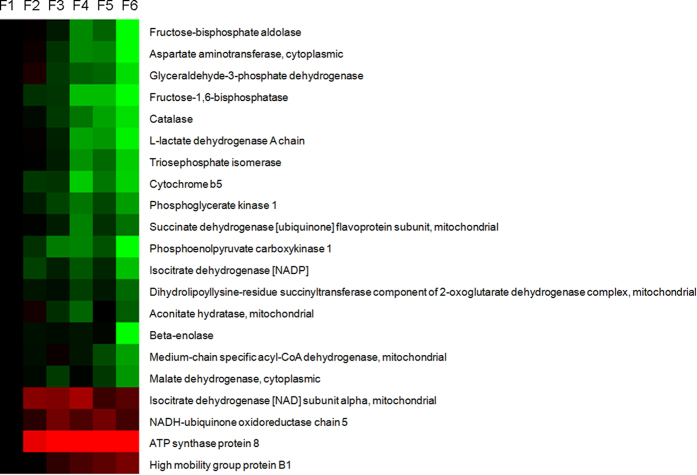
The expression of proteins in glycolysis along CVA. The proteins in glycolysis pathway were selected based on the results of KEGG pathways enrichment. The mRNA expression of genes and the activities of enzymes involved in glycolysis were measured using RT-PCR and commercial kits, respectively.

As seen in Fig. 5, the expression of most of the identified proteins involved in glycolysis/gluconeogenesis was down-regulated from F1 to F6. Then we tested the mRNA expression and activities of some key enzymes involved in glycolysis. The results of RT-PCR also showed down-regulated mRNA expression of phosphofructokinase and pyruvate kinase from F1 to F6. The activities of phosphofructokinase, pyruvate kinase and pyruvate dehydrogenase in the epithelial cells with different degree of differentiation were measured, and the activity of phosphofructokinase gradually decreased from F1 to F6, and the activities of pyruvate kinase and pyruvate dehydrogenase were also decreased from villi to crypt.

### Fatty acids metabolism

**Figure 6:**
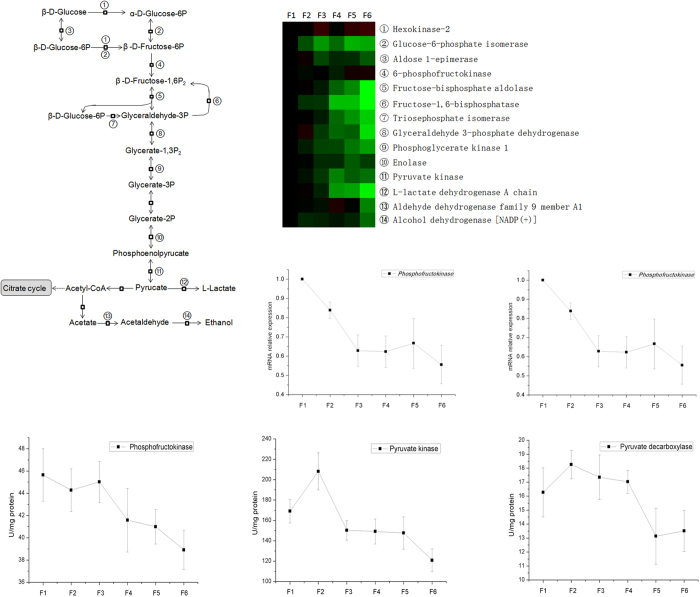
The expression of proteins in β-oxidation along CVA. The proteins in β-oxidation were selected based on the results of KEGG pathways enrichment. The mRNA expression of peroxisome proliferator-activated receptors (PPARs) and the contents of free fatty acids were measured using RT-PCR and commercial kits, respectively.

Fatty acid β-oxidation is a multi-step process by which fatty acids are broken down to produce energy. As seen in Fig. 6, eight proteins involved in β-oxidation were identified and the expression of most of the proteins decreased from F1 to F6. The contents of free fatty acids in the epithelial cells with different degree of differentiation were tested, and the contents were greater in villus cells than crypt cells. Peroxisome proliferator-activated receptors (PPARs) are transcription factors belonging to nuclear receptors superfamily andhave three isoforms (α, β/δ, and γ), which involved in regulating fatty acid oxidation^21^. The mRNA expression of PPAR-α, PPAR-β/δ, and PPAR-γ was tested by RT-PCR, and their expression in villus cells was greater than crypt cells.

### Animo aicds metabolism

**Figure 7:**
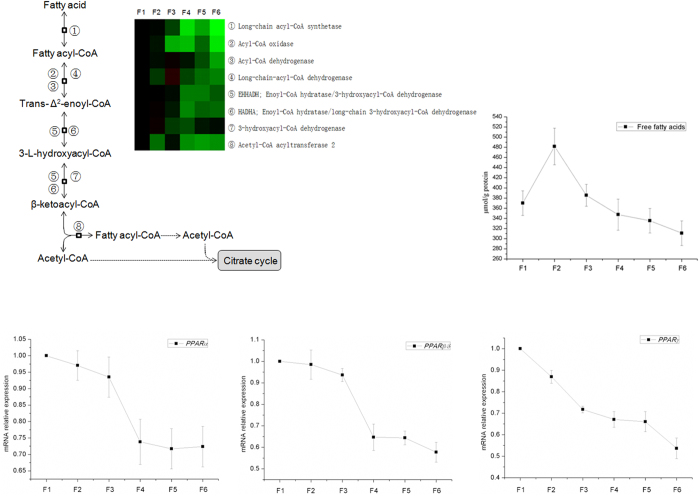
The expression of proteins related to amino acids metabolism along CVA. The proteins in amino acids metabolism were selected based on the results of functional categorization. The activities of enzymes involved in amino acids metabolism were measured using commercial kits.

A total of 16 proteins involved in amino acids metabolism were identified (Fig. 7). The expression of most of the proteins were down-regulated from F1 to F6 excepting asparagine synthetase, dihydrofolate reductase, and glutaminase, whose expression increased from F1 to F6 (Fig. 7). Then we tested the activities of 2 key enzymes (glutamic-pyruvic transamine and glutamic-oxalacetic transamine) involved in amino acids metabolism. The activities of glutamic-pyruvic transamine and glutamic-oxalacetic transamine were decreased from F1 to F6 (Fig. 7).

### Citrate cycle

**Figure 8:**
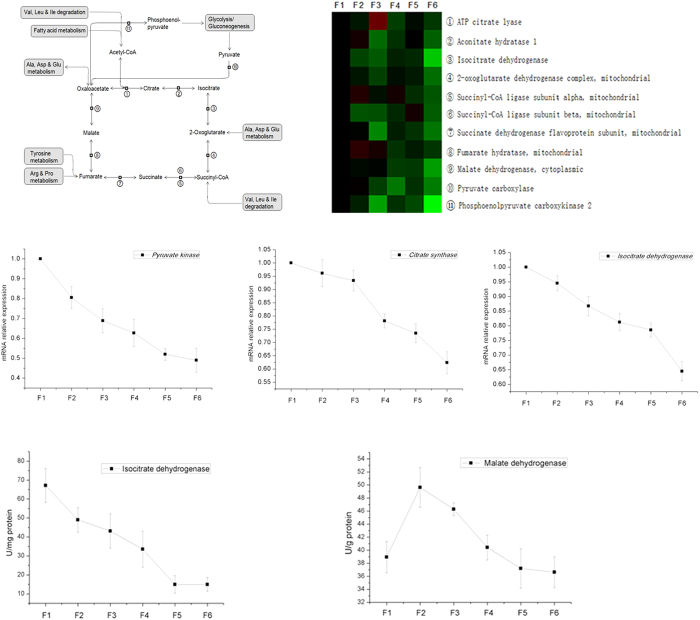
The expression of proteins in citrate acid cycle along CVA. The proteins in citrate acid cycle were selected based on the results of KEGG pathways enrichment. The mRNA expression of genes and the activities of enzymes involved in tricarboxylic acid cycle were measured using RT-PCR and commercial kits, respectively.

A total of 11 proteins involved in citrate cycle were identified, and the expression of most of the identified proteins was down-regulated from F1 to F6 (Fig. 8). Then we tested the mRNA expression and activities of some key enzymes involved in citrate cycle. The mRNA expression of citrate synthase, isocitrate dehydrogenase, and oxoglutarate dehydrogenase were also down-regulated from F1 to F6 (Fig. 8). The activities of isocitrate dehydrogenase and malate dehydrogenase were measured, the activity of isocitrate dehydrogenase gradually decreased from F1 to F6, the activity of malate dehydrogenase in villus cells were also greater than in crypt cells (Fig. 8).

### Oxidative phosphorylation

**Figure 9:**
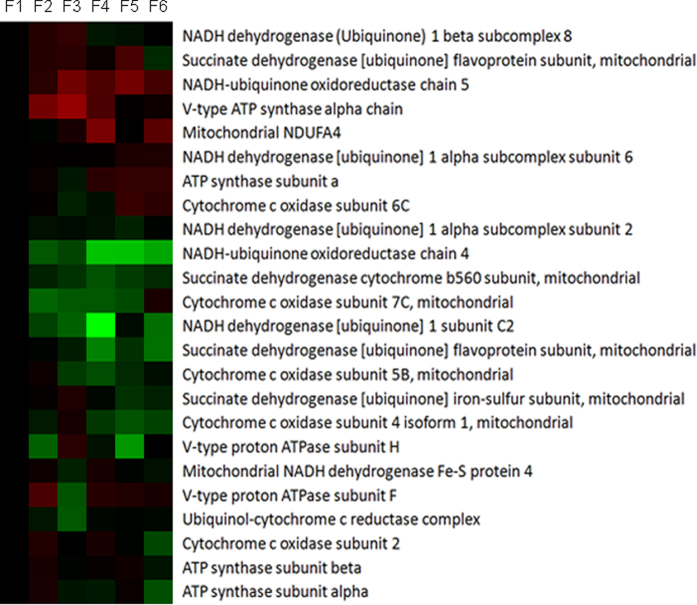
The expression of proteins in oxidative phosphorylation along CVA. The proteins in cell cycle and apoptosis were selected based on the results of KEGG pathways enrichment.

The proteins involved in oxidative phosphorylation were also enriched by KEGG database. A total of 24 proteins involved in oxidative phosphorylation were identified from the differentially expressed proteins. The results showed that some of proteins with a role in oxidative phosphorylation were down-regulated from F1 to F6, and some of proteins were up-regulated. The expression of proteins related to succinate dehydrogenase and cytochrome c oxidase was mainly down-regulated from F1 to F6 (Fig. 9).

### Intestinal morphology

**Figure 10:**
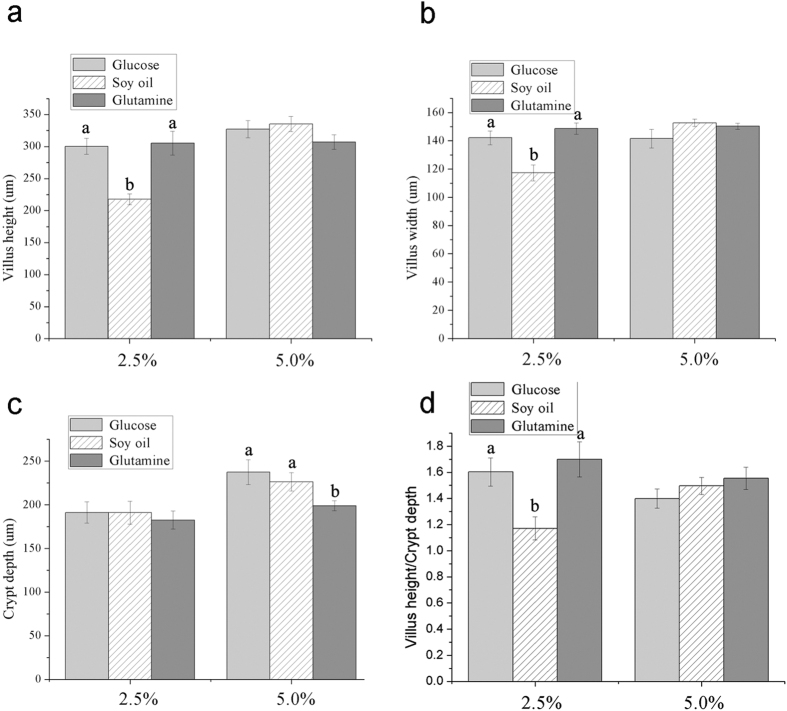
The effects of energy sources on intestinal morphology of weaned piglets. The piglets were weaned at 21 d of age and fed diets with 2.5% or 5% of the total energy provided by glucose, soy oil or glutamine for a 14-d period. The intestinal morphology of jejunum was measured.

Compared with piglets with 2.5% of the total energy provided by glucose and glutamine, the villus height and villus width of piglets that were given 2.5% of the total energy by soy oil were reduced (Fig. 10a,b). There were no significant differences in villus height and villus width among piglets with 5% of the total energy provided by glucose, soy oil, and glutamine (Fig. 10a,b). The crypt depth of piglets with 5% of the total energy provided by glutamine was lower than that of piglets with 5% of the total energy provided by glucose and soy oil (Fig. 10c). No significant differences in crypt depth among piglets with 2.5% of the total energy provided by glucose, soy oil, and glutamine were observed (Fig. 10c). The villus height/crypt depth of piglets with 2.5% of the total energy provided by soy oil was lower than that of piglets with 2.5% of the total energy provided by glucose and glutamine (Fig. 10d). No significant differences in villus height/crypt depth among piglets with 5% of the total energy provided by glucose, soy oil, and glutamine were observed (Fig. 10d).

## Discussion

The intestinal epithelial cells undergo continual renewal that involves highly coordinated processes of cellular proliferation, differentiation, migration, and apoptosis along CVA^22^. These processes of intestinal epithelial cells along CVA are accompanied by physiological phenotypic and functional changes, which are realized by the expression of intestinal epithelial cells specific proteins. The present study provides a global protein expression profile in intestinal epithelial cells during maturation along CVA in piglets. A lot of changes were observed during the maturation process, including cellular process, metabolic process, biological regulation, pigmentation, multicellular organizational process, and response to stimulus and so on. Fan *et al.*^18^ reported that the activities of ALP, aminopeptidase N, sucrose, Lactase, and Na^+^/K^+^ -ATPase in jejuna and ileal epithelial cells of piglets increased during maturation along CVA^18^. The capacity of intestinal epithelial cells in L-glutamate, L-glutamine, and glucose uptake also increased during maturation along CVA in piglets^19^,^23^. The digestion and absorption is the process that is critically depend on energy, the portal-drained viscera (PDV; including intestines, pancreas, spleen, and stomach) contribute less than 6% of body weight, but contribute 20–35% of whole-body energy expenditure^1^,^3^. Accompanied with increased digestive enzymes activities and nutrients uptake capacity, proteins related to mitochondrion and energy metabolism in intestinal epithelial cells also increased during maturation along CVA in piglets.

Intestinal mucosa energy metabolism is very complex, because the energy source for intestinal mucosa is a complex mixture of arterial and luminal substrates and the pattern of intestinal substrates oxidation can be altered by nutrients composition in the diets^4^. Stoll *et al.*^5^ showed that enteral glucose and arterial glucose contributed to 15% and 29% of the total production of CO_2_ in the PDV of piglets, respectively^5^. The contents of glucose in villus tip and the bottom of the crypt were greater than that in the middle of villi in piglets (unpublished data). These results indicated that the intestinal epithelial cells used both enteral and arterial glucose. Moreover, kinetic parameter estimates of Na^+^-D-glucose co-transport into apical membrane vesicles showed that the maximal transport rate and SGLT1 affinity of glucose in middle villus and crypt epithelial cells were greater than upper villus epithelial cells, and the apparent transmembrane diffusion rate of glucose in crypt epithelial cells was also greater that in upper villus epithelial cells^20^. Chang *et al.*^11^ reported that proteins involved in glycolysis were up-regulated in adult mouse small intestinal villus epithelial cells compared with crypt epithelial cells^11^. Consistent with it, the results of the present showed that the expression of proteins involved in glycolysis were also increased in piglet jejunal epithelial cells during maturation along CVA. Moreover, the mRNA expression of genes and the activities of enzymes related to glycolysis in jejunal epithelial cells were also increased from the bottom of crypt to the tip of villi. These results indicated that the catabolism of glucose in intestinal epithelial cells of neonatal animals were increased during maturation along CVA.

Besides glucose, amino acids, especially glutamate and glutamine, also play important roles in providing energy to intestinal epithelial cells^24^. Although the PDV tissues only account for about 6% of body weight, they are responsible for more than 50% of the whole-body turnover of some essential amino acids^2^. Windmueller and Spaeth (1975, 1976) demonstrated extensive utilizations of glutamine and glutamate by small intestine, and about 66% and 98% of luminal glutamine and glutamate was catabolized by rat jejunum in a single pass, respectively^25^,^26^. Stoll *et al.*^5^ also showed that arterial glutamine and enteral glutamate contributed to 19% and 36% of the total production of CO_2_ in the PDV of piglets, respectively^5^. The results of the present experiment showed that the expression of proteins involved in amino acids metabolism were mainly increased in intestinal epithelial cells during maturation along CVA, excepted the proteins (asparagine synthetase, dihydrofolate reductase, and glutaminase) involved in glutamine metabolism. Moreover, the activities of glutamic-pyruvic transamine and glutamic-oxalacetic transamine were also increased from crypt to upper villus epithelial cells. These results indicated that the villus epithelial cells have greater metabolism in most of amino acids, while the crypt epithelial cells have a greater metabolism in glutamine. It was showed that the metabolism of dietary glutamate was more extensive than that of enteral glutamine^27^, which may be because the villus epithelial cells have higher glutamate uptaking and metabolism capacities. Windmueller and Spaeth (1975) indicated that the present of high contents of glutamate in the intestinal lumen had a very small (about 25%) effects on the utilization of glutamine by intestine^25^, from the results of the present experiment, it may be because the glutamate and glutamine were metabolized in different epithelial cells and they may have different functions. Moreover, the glutamine metabolized by crypt epithelial cells may mainly uptake from the mesenteric artery as about 30% of the artery glutamine was extracted in a single pass by the small intestine of rat^25^,^26^.

Although the primary function of small intestine in lipid metabolism is triacylglycerol (TG) resynthesis and the secretion of TG as chylomicrons, β-oxidation related enzymes were also expressed in the small intestine and their expression could be regulated by dietary supplementation with oils^28^. These results indicated that the small intestine was involved in fatty acids metabolism and fatty acids may be one of the energy sources for intestine. Previous experiments showed an increase in the mRNA expression of genes involved in lipid metabolism in villi compared with that in crypt in the intestine of aldult mouse^12^,^16^. The contents of lipid metabolites were also greater in villi compared with that in crypts^12^. In agreement with it, the results of the present experiment showed that the expression of proteins involved in β-oxidation were increased in intestinal epithelial cells during maturation along CVA in piglets. Moreover, the concentrations of free fatty acids and mRNA expression of PPARs were also greater in villus epithelial cells than that in crypt epithelial cells. These results indicated that the villus epithelial cells had greater fatty acids catabolism than crypt epithelial cells, which may be resulted from a grater fatty acids uptaking capacity in villus epithelial cells than crypt epithelial cells^16^.

The citrate cycle plays a key role in the oxidation of various substrates, such as glucose, fatty acids, and amino aicds (glutamine, glutamate, and aspartate)^29^,^30^. Consistent with the increase in glucose, amino acids, and fatty acids metabolism in intestinal epithelial cells during maturation along CVA, the expression of proteins involved in citrate cycle also increased from the bottom of crypt to the top of villi in jejunum of piglets. The mRNA expression of genes and the activities of enzymes related to citrate cycle also increased from crypt to villi. Although the expression trend of proteins involved in oxidative phosphorylation in intestinal epithelial cells during maturation along CVA were not consistent, the expression of proteins related to succinate dehydrogenase and cytochrome c oxidase were mainly increased from crypt to villus tip. Further researches are needed to study the oxidative phosphorylation in intestinal epithelial cells during maturation along CVA. These results indicated that energy metabolism in intestinal epithelial cells was mainly increased during maturation along CVA and the intestinal epithelial cells with different maturation levels may have different nutrients requirement in piglets. Therefore, dietary supplementation with different nutrients may have different effects on the renewal of intestinal epithelial cells. Weaning is one of the most stressful events the pigs encounter in modern swine production, which results in intestinal morphology changes and dysfunction^31^. The most marked changes in intestinal structure in weaning piglets are the increases in crypt depth and reductions in villus height^7^. The results of the present experiment showed that the villus height, villus width, and villus height/crypt depth in weaned piglets with 2.5% of the total energy provided by glucose and glutamine were greater than that in piglets with 2.5% of the total energy by soy oil, which indicated that the intestinal structure was affected by energy sources. Moreover, the crypt depth of weaned piglets with 5% of the total energy provided by glutamine were reduced compared with piglets with 5% of the total energy provided by glucose or soy oil, which may be because the energy of crypt epithelial cells were mainly provided by glutamine and dietary supplementation with a high dose of glutamine improved intestinal structure of weaned piglets by reducing crypt depth. Overall, intestinal epithelial cells with different maturation levels had different energy sources and dietary supplementation with different energy sources had different effects on intestinal morphology.

In conclusion, results of the present experiment show a global change in protein expression in intestinal epithelial cells during maturation along CVA in piglets. The energy metabolism in intestinal epithelial cells of piglets was increased from the bottom of crypt to the top of villi. Moreover, the expression of proteins related to metabolism of glucose, most of amino acids, and fatty acids was increased in intestinal epithelial cells during maturation along CVA, while the expression of proteins related to glutamine metabolism was decreased from crypt to villus tip. The expression of proteins involved in citrate cycle was also increased intestinal epithelial cells during maturation along CVA. In addition, dietary supplementation with different energy sources had different effects on intestinal structure of weaned piglets. These results will provide potential methods to use energy sources to regulate intestinal morphology and functions.

## Materials and Methods

### Reagents

DL-β-Hydroxybutyrate sodium salt was purchased from J & K Chemical (Ltd., USA). Trypsin was procured from Promega (Madison, WI, USA). iTRAQ-reagent was purchased from Applied Biosystems (Foster City, CA, USA). Bovine serum alumin (BSA, fraction V), phenylmethylsulfonyl fluoride (PMSF), dithiothreitol (DTT), and other chemical were obtained from Sigma-Aldrich (St. Louis, MO, USA) unless otherwise stated.

### Sequential isolation of epithelial cells along CVA

A total of eight suckling piglets (21 days of age) were purchased from the Hunan Institute of Animal Husbandry and Veterinary Medicine (Changsha, China). Piglets were maintained under general anesthesia and sacrificed by an intravenous (jugular vein) injection of 4% sodium pentobarbital solution (40 mg/kg born weight). Intestinal epithelial cells were isolated using the distended intestinal sac method as previously described with slight modifications^18^. The divided mid-jejunum segments were rinsed thoroughly with ice-cold physiological saline solution and incubated at 37 °C for 30 min with oxygenated PBS. Then, six “cell fractions” (designated F1 through F6) were collected along CVA using oxygenated isolation buffer (5 mM Na2EDTA, 10 mM HEPES pH 7.4, 0.5 mM DTT, 0.25% BSA, 2.5 mM D-glucose, 2.5 mM L-glutamine, 0.5 mM dl-β-hydroxybutyrate sodium salt, oxygenated with an O2/CO2 mixture (19:1, v/v)). Each of the F1 and F2 fractions were collected after a separate 20 min incubation, F3 and F4 were collected after a separate 25 min incubation, and F5 and F6 were collected after a separate 30 min incubation. Each of the cell fraction was washed twice with oxygenated cell resuspension buffer (10 mM HEPES, 1.5 mM CaCl2, 2.0 mM MgCl2, pH 7.4), and centrifuged at 400 × g for 4 min at 4 °C. The isolated cells were immediately frozen in liquid nitrogen and then stored at −80 °C until analysis. The experimental design and procedures used in this study were carried out in accordance with the Chinese Guidelines for Animal Welfare and Experimental Protocols, and approved by the Animal Care and Use Committee of the Institute of Subtropical Agriculture at the Chinese Academy of Sciences.

### Sample preparation and isobaric labeling

The harvested cells were re-suspended and disrupted in lysis buffer composed of 7 M urea, 2 M thiourea, 4% w/v CHAPS, 20 mM TBP, 0.2% Bio-lyte (pH 3-10), and a protease inhibitor cocktail (Roche Diagnostics Ltd, Mannheim, Germany). DNAse I and RNAse A were added to the lysate at final concentrations of 1 mg/mL and 0.25 mg/mL, respectively. After cell disruption, the protein solution was separated from the cell debris by centrifugation (12,000 × g, 5 min, 4 °C). The crude protein extracts were further purified using the Ready Prep 2-D Cleanup Kit (Bio-Rad Laboratories, USA) and then underwent a reductive alkylation reaction. The protein concentration was determined using a 2-D Quant Kit (GE Healthcare, USA). Trypsin digestion and iTRAQ labeling were performed according to the manufacturer’s protocol (Applied Biosystems, Foster City, CA, USA). Briefly, 100 μg total protein of cell fraction was reduced and alkylated, digested overnight at 37 °C with trypsin (Promega, Madison, WI, USA), and labeled with iTRAQ-reagents (Applied Biosystems, Foster City, CA, USA) as follows: F1 (villus tip), iTRAQ reagent 113; F2 (upper villi), iTRAQ reagent 114; F3 (middle villi), iTRAQ reagent 115; F4 (middle villi), iTRAQ reagent 116; F5 (upper crypt region), iTRAQ reagent 117; F6 (crypt bottom), iTRAQ reagent 118.

### Peptide fractionation and LC-MS/MS acquisition

The isotopically labeled samples were pooled and fractionated by strong cation exchange chromatography on an LC-20AD HPLC system (Shimadzu Scientific Instruments, Kyoto, Japan), using a polysulfoethyl A column (2.1 × 100 mm, 5 μm, 300 Å; The Nest Group Inc., Southborough, MA, USA), as previously described^32^. The eluted fractions were desalted using a Sep-Pak C18 Cartridge (Waters, Milford, MA, USA) and diluted with the loading buffer (10 mM KH2PO4 in 25% acetonitrile, pH 2.8). Buffer A was identical, in composition, to the loading buffer, and buffer B was buffer A with 350 mM KCl. Separation of proteins was performed using a linear binary gradient of 0–80% buffer B in buffer A at a flow rate of 200 μL/min for 60 min. The absorbance at 214 nm and 280 nm was monitored, and a total of 30 fractions were collected along the gradient.

Each elution fraction from the polysulfoethyl A column was dried down, dissolved in buffer C (5% acetonitrile, 0.1% formic acid), and analyzed on QStar XL (Applied Biosystems), as previously described^33^. Briefly, peptides were separated on a reversed-phase (RP) column (ZORBAX 300SB-C18 column, 5 μm, 300 Å, 0.1 × 15 mm; Waters) using an LC-20AD HPLC system (Shimadzu Scientific Instruments). The HPLC gradient was 5–35% buffer D (95% acetonitrile, 0.1% formic acid) in buffer C at a flow rate of 0.2 μL/min for 65 min. Survey scans were acquired from m/z 400–1800 with up to four precursors selected for MS/MS from m/z 100–2000 using a dynamic exclusion of 30 s. The iTRAQ labeled peptides fragmented under collision-induced dissociation conditions to give reporter ions at m/z 117.1, 118.1, 119.1, and 121.1. The ratios of the peak areas of the iTRAQ reporter ions reflect the relative abundances of the peptides and, consequently, the proteins in the samples. Larger, sequence-information-rich fragment ions were also produced under these MS/MS conditions, providing the identity of the protein from which the peptide originated.

### Data analysis and quantification

Database searches to identify the peptides were performed with ProteinPilot™ 3.0 software (Software rev. 114732; Applied Biosystems), using the Paragon and ProGroup algorithms against the Uniprot non-redundant database, which contained information on pig proteins. The precursor tolerance was set to 0.2 Da, and the iTRAQ fragment tolerance was set to 0.2 Da. The search parameters were: i) enzyme cleavage by trypsin with one missed cleavage allowed and ii) the fixed modifications of MMTS were set as Cys. An automatic decoy database search strategy was employed to estimate the false discovery rate (FDR). The FDR for the protein identification was calculated by searching against a concatenated reversed database. In the final search results, the FDR was less than 1.5%. The protein confidence threshold cutoff was 1.3 (Unused ProtScore) from at least two peptides with 95% confidence. For protein identification, the filters were set as follows: significance threshold P ≤ 0.05 (with 95% confidence) and ion score or expected cutoff ≤0.05 (with 95% confidence). In present study, a protein with ≥ 1.2-fold or ≤0.8 -fold difference and a P-value ≤ 0.05 was regarded as being differentially expressed (Supplemental dataset).

### Bioinformatics analysis

The KEGG data base and the WEGO program were used to classify and group the differentially expressed proteins^34^. The cluster of differentially expressed proteins was done by Cluster 3.0 using k-means clustering (http://bonsai.hgc.jp/~mdehoon/software/cluster/software.htm) and the comparison of up-regulated (Up) and down-regulated (Down) proteins from F1 (villus tip) to F6 (crypt bottom) was performed by WEGO program^34^.

### Enzyme activity and Western Blot analysis

The activities of alkaline phosphatase, phosphofructokinase, pyruvate kinase, pyruvate dehydrogenase, glutamic-pyruvic transaminase, glutamic-oxalacetic transaminase, isocitrate dehydrogenase, and malate dehydrogenase, and the concentrations of free fatty acids were determined using commercial kits according to the manufacturer’s protocol (Alkaline phosphatase assay kit was purchased from Nanjing Jiancheng Bioengineering Institute, Nanjing, China; Others were purchased from Suzhou Comin Biotechnology Co., Ltd, Suzhou, China). Western blot analysis was performed as previously described^35^. The denatured proteins of cell fractions were separated using SDS-PAGE (10% gradient gel), and transferred to PVDF membranes (Millipore, Billerica, MA) overnight at 12 V using a Bio-Rad Transblot apparatus (Hercules, CA). The membranes were blocked in 5% fat-free milk in Tris-Tween buffered saline (TTBS: 20 mM Tris/150 mM NaCl, pH 7.5, and 0.1% Tween-20) for 3 h and then incubated with primary antibodies at 4 °C overnight with gentle rocking. After being washed three times with TTBS, the membranes were incubated at room temperature for 2 h with horseradish peroxidase-linked secondary antibodies. Finally, the membranes were washed with TTBS, and then developed using Supersignal West Dura Extended Duration Substrate according to the manufacturer’s instructions (Pierce, Rockford, IL). The images were detected by exposing the blots to X-ray film in a cassette. Western blots were quantified by measuring the intensity of correctly sized bands using AlphaImager 2200 (Alpha Innotech Corporation, CA, USA) software. The antibodies for β-actin and PCNA were purchased from Cell Signaling Technology and Santa Cruz (CA, USA), respectively.

### RNA extraction and RT-PCR

Total RNA was purified from the isolated epithelial cells of piglets using TRIZOL reagent (Invitrogen, CA, USA) and treated with DNase I (TaKaRa, Dalian, China), according to the manufacturer’s instructions. Then cDNA was reverse transcribed using the RevertAcidTM first strand cDNA synthesis kit (TaKaRa). Oligo 6.0 software (Molecular Biology Insights, CO, USA) was used to design primers for phosphofructokinase, pyruvate kinase, PPAR α, PPAR β/δ, PPARγ, citrate synthase, isocitrate dehydrogenase, oxoglutarate dehydrogenase (Table 1). Real-time quantitative PCR (RT-PCR) analyses were performed using ABI 7900HT Fast Real-Time PCR System (Applied Biosystems, Carlsbad, CA). Each PCR reaction with a total volume of 10 μL containing 5 μL SYBR Green mix, 1 μL 4 × diluted cDNA, 0.2 μL each of forward and reverse primers. and 0.2 μL ROX Reference Dye (50×). After a pre-denaturation process (10 s at 95 °C), a total of 40 cycles (each cycle was consisted of 95 °C for 5 s and 60 °C for 20 s) of amplification were conducted, which was followed by a melting curve program (from 60 °C to 99 °C with a heating rate of 0.1 °C/s, and the fluorescence was collected). In each sample, the *β-actin* was used as reference gene to normalize the expression of the interested genes. The relative mRNA expression ratio (R) of each interested gene was calculated by *R* = 2^−ΔΔ*Ct*(*sample* −^ ^*control*)^, where −ΔΔ*Ct*(*sample* − *control*) = (*Ct gene of interest* − *Ct of β-actin*)_*sample*_ − (*Ct gene of interest* − *Ct of β-actin*)_*control*_. The efficiency of RT-PCR was determined by amplifying a dilution series of cDNA according to the equation 10(−1/slope). The interested mRNA and *β-actin* mRNA were amplified with comparable efficiencies. In negative controls, the cDNA sample was replaced by water.

### Animal and intestinal morphology

A total of 48 Duroc × Landrace × Yorkshire piglets were weaned at 21 d of age, and were blocked by body weight, sex, and litter and randomly assigned to 1 of 6 treatments, with 2.5% or 5% of the total energy was provided by glucose, soy oil or glutamine for a 14-d period. At 35 d of age, piglets were anesthetized (with C3H2ClF5O) and killed by injecting jugular vein with 4% sodium pentobarbital solution (40 mg/kg BW). Then, the gastrointestinal tract of killed piglets was removed immediately and dissected. Approximately 2 cm of intestinal segments were aseptically isolated from the middle sections of jejunum, and then flushed using phosphate-buffered saline and fixed using a 10% formaldehyde–phosphate buffer. The fixed intestinal segments were embedded using low-melt paraffin wax. Three cross sections of each intestinal segment were 5-μm in thickness, and stained with hematoxylin and eosin. Villus height and crypt depth of each intestinal segment were measured at 40× magnification using image processing and an analysis system (version 1, Leica Imaging Systems Ltd., Cambridge, UK). At least ten well-oriented intact villi and its associated crypt were determined in each intestinal section of each piglet. The mean of villus height and crypt depth of each section was then calculated per piglet and used for further analysis. The data were subjected to one-way analysis of variance (ANOVA) using SAS software (Version 9.2; SAS Institute Inc., Cary, NC). Probability values < 0.05 were used to indicate statistical significance among means.

## Additional Information

**How to cite this article**: Yang, H. *et al.* Energy metabolism in intestinal epithelial cells during maturation along the crypt-villus axis. *Sci. Rep.* **6**, 31917; doi: 10.1038/srep31917 (2016).

## Supplementary Material

Supplementary Dataset

## Figures and Tables

**Table 1:** Primer sequence for PCR.

Genes	Sequences (50–30)	Genebank accession	Product size
*PFK*	TGGATGGCGGAGATAACA	NM_001044550.1	221 bp
	CCACTCAGAGCGGAAGGT		
*PYK*	TCGCATCTTTCATCCGTAA	XM_005666189.1	124 bp
	TCATCAAATCTCCGAACTCC		
*PPARα*	TCAAGAGCCTGAGGAAACC	DQ437887.1	121 bp
	CAAATGATAGCAGCCACAAA		
*PPARβ/δ*	CAGAGCACTCGCTTCCCT	NM_214152.2	152 bp
	GCCTGATGCCTTGTCCC		
*PPARγ*	GCAGGAGCATAGCAAAGAG	DQ437884.1	58 bp
	GGAGCGAAACTGACACCC		
*CISN*	ATGAAGGTGGCAATGTAAG	NM_214276.1	228 bp
	CCCGTCCTGAGTTGAGTG		
*ICDH*	ATTCTGAAAGCCTACGACG	NM_001164007.1	143 bp
	GAAGACTTGAGGACCTGAGC		
*OxoGDH*	CGTGACCGACAGGAACATC	XM_003134891.4	239 bp
	CGTGGACAGTGCCGTGAG		
*β-actin*	TGCGGGACATCAAGGAGAAG	XM_003357928.2	216 bp
	AGTTGAAGGTGGTCTCGTGG		

PFK, Phosphofructokinase; PYK, Pyruvate kinase; PPARα, peroxisome proliferator-activated receptor α; PPARβ/δ, peroxisome proliferator-activated receptor β/δ; PPARγ, peroxisome proliferator-activated receptor γ; CISN, citrate synthase; ICDH, isocitrate dehydrogenase; OxoGDH, oxoglutarate dehydrogenase.
